# Associations between therapist factors and treatment efficacy in randomized controlled trials of trauma‐focused cognitive behavioral therapy for children and youth: A systematic review and meta‐analysis

**DOI:** 10.1002/jts.22840

**Published:** 2022-04-27

**Authors:** Lauren Grainger, Zoe Thompson, Nexhmedin Morina, Thole Hoppen, Richard Meiser‐Stedman

**Affiliations:** ^1^ Department of Clinical Psychology and Psychological Therapies, Norwich Medical School University of East Anglia Norwich United Kingdom; ^2^ Department of Psychology University of Münster Münster Germany

## Abstract

Previous research suggests that the effect of therapist factors on patient outcomes is significant. Yet, to date, no reviews have explored the potential effects of therapist characteristics on treatment outcomes for children and youth with posttraumatic stress disorder (PTSD). This systematic review and meta‐analysis aimed to summarize the professional characteristics of trial therapists delivering trauma‐focused cognitive behavioral interventions (TF‐CBT) for child PTSD in clinical trials and understand the association between treatment efficacy and therapist factors. Systematic searches for randomized controlled trials (RCTs) published through November 3, 2020, were conducted; 40 RCTs were included in the full review. PTSD treatment outcome data were extracted from each publication along with any available data regarding trial therapists. Subgroup analyses were conducted to compare the outcomes of interventions conducted by different types of therapists. All therapist groups yielded significant effects for TF‐CBT relative to active and passive control conditions, with the largest effect size, Hedges’ *g* = −1.11, for RCTs that used clinical psychologists and psychiatrists. A significant moderating effect was found when comparing the treatment outcomes of clinical psychologists and psychiatrists versus other professionals, *p* = .044; however, this effect was no longer apparent when only studies with an active control arm were included. Further moderator analyses demonstrated no significant differences regarding therapists’ educational and professional backgrounds and PTSD treatment outcomes. The current RCT evidence for TF‐CBT for children and youth with PTSD does not suggest that therapist educational or professional background influences treatment efficacy. Limitations and implications for future research are discussed.

The impact of therapist factors on treatment outcomes for common mental health difficulties has been previously documented but is not yet fully understood (Castonguay & Hill, 2017; Norcross & Lambert, 2019; Wampold & Imel, 2015). Previous research, although mainly conducted in adult populations, suggests that the clinical implications of therapist factors on patient outcomes are significant and affect many areas of patient experience. For example, patient dropout rates have been found to be 4 times higher among patients treated by less effective therapists (Saxon et al., 2017), and patient recovery rates of the most effective therapists have been shown to be twice as high as those achieved by the least effective therapists (Okiishi et al., 2003).

A number of therapist variables have been investigated, including therapist theoretical orientation (Anderson et al., 2009; Bergler et al., 2016; Okiishi et al., 2006), age (Anderson et al., 2009; Shauenburg et al., 2005), and gender (Shiner et al., 2017; Zorzella et al., 2015), but results from these studies have frequently contradicted one another. For example, therapist competency has been found to be a predictor of symptom change in treatments for depression (Strunk et al., 2010) and was found to account for 48% of the variance in patient outcomes for social anxiety disorder (Ginzburg et al., 2012). However, in a meta‐analysis conducted by Webb and colleagues (2010), there appeared to be no association between therapist competence and patient outcomes. These mixed findings highlight the need for further investigation into the impact of therapist variables on patient outcomes. In a recent review, Johns et al. (2019) concluded that ongoing investigations of therapist effects are vital so that the characteristics of effective therapists can be identified and more efficient matching of therapist and patient can be facilitated. Unfortunately, many clinical trials fail to report therapist variables in much detail, thus limiting the breadth of reviews that can be conducted within this area and making it difficult to draw firm conclusions about therapist variables and treatment efficacy.

An area of growing clinical interest in the literature concerns evidence‐based therapies for children and youth who are experiencing posttraumatic stress disorder (PTSD). PTSD is a highly distressing psychiatric disorder characterized by the pervasive reliving of traumatic events through flashbacks and nightmares, as well as hypervigilance to threats and the avoidance of any reminders of a traumatic experience (American Psychiatric Association, 2013). With surveys suggesting that only 1 in 5 young people with PTSD in the United Kingdom has accessed support from a mental health professional (Lewis et al., 2019), there is a growing demand for services that provide effective and timely treatments. Guidelines created by both the National Institute for Health and Care Excellence guidelines (2018) and the International Society for Traumatic Stress Studies (2018) recommend that the primary treatment type for trauma‐exposed youth should be psychological therapy, particularly trauma‐focused cognitive behavioral therapy (TF‐CBT).

Previous research has noted that trauma‐focused therapies are an area where therapists commonly feel they lack competence and experience, which can be a barrier to evidence‐based practice (Finch, Ford, Grainger, &, Meiser‐Stedman, 2020). Despite this, few reviews have focused on TF‐CBT therapists, with fewer still in child populations (Baldwin & Imel, 2013; Johns et al., 2019; Fjermestad et al., 2016). In their review, Pfeiffer and colleagues (2020) collated therapist data from two randomized controlled trials (RCTs) examining the effectiveness of TF‐CBT for children and youth and found no evidence of an association between therapists’ theoretical background or clinical experience and patient outcomes. Although there is a significant evidence base for trauma‐focused therapies for children and youth, particularly for TF‐CBT, Pfeiffer and colleagues’ (2020) review is, to our knowledge, the only study to date that has examined the impact of therapist factors in TF‐CBT for this population. However, no reviews have yet provided a summary of the providers delivering TF‐CBT in the research trials or the clinical guidelines on which the trials are based, therefore making it difficult to understand the effect of therapist factors on trial outcomes more widely. Some researchers have argued that the importance of identifying therapist factors that promote effective treatment outcomes is particularly important in youth populations, as, typically, young people are less likely to refer themselves for therapy than adults, with parents or guardians instead making these referrals (Podell et al., 2013). Youth, therefore, may look more to the therapeutic relationship for motivation to engage in therapy.

In addition, globally, a large treatment gap exists between individuals who would benefit from mental health care interventions and those who receive such care (Singla et al., 2017). The number of individuals with PTSD is particularly high in low‐ and middle‐income countries (LMICs) with a recent history of war (Hoppen et al., 2021). A significant barrier to receiving psychological treatment in LMICs is the huge scarcity of skilled human resources (Saxena et al., 2007). Consequently, many individuals have advocated for the task‐shifting of mental health interventions to lay health workers to provide psychological interventions for pediatric PTSD (Singla et al., 2017). This approach has been investigated in several clinical trials with children and adolescents with PTSD (e.g., Dorsey et al., 2020; Ertl et al., 2011). Systematically assessing the efficacy of the interventions delivered by lay professionals relative to the provision by mental health professionals remains of great significance. The present review aimed to examine which therapist variables are most commonly reported within the area of child PTSD research and investigate their links to treatment efficacy.

## METHOD

### Search procedure and quality assessment

The review was prospectively registered on PROSPERO register of systematic reviews (June 11, 2020; CRD42020218106). The present study utilized and updated previous searches conducted by Hoppen and Morina (2020) and Morina et al. (2016). The utilization of this methodology felt most appropriate due to the present study's focus on the RCT data utilized to inform clinical practice guidelines. Given the exclusive focus of the current meta‐analysis on TF‐CBT interventions, only studies that included this kind of intervention in at least one arm of a trial were included. The term TF‐CBT encompassed cognitive behavioral interventions with a focus on trauma memories, such as cognitive therapy and prolonged exposure. If a publication reported two active TF‐CBT treatment groups without a third control condition, it was excluded due to its lack of a meaningful control arm. To be considered eligible, trials had to meet the following inclusion criteria: (a) a random allocation of participants, (b) at least one arm with a TF‐CBT intervention for child and adolescent PTSD in comparison to a passive (i.e., no active intervention provided) or active (i.e., alternative intervention or treatment provided) control group or to another psychological intervention, (c) an average participant age below 18 years in the full sample, and (d) at least 10 participants per group at posttreatment and/or follow‐up. In line with previous protocol, trials that examined interventions that were not specifically designed as PTSD treatment interventions, such as classroom‐based interventions, were excluded.

Searches completed by Hoppen and Morina (2020) were updated in May 2021 to include publications since 2019, using similar search criteria and the same databases (i.e., MEDLINE, PsycINFO, and Web of Science). The following terms were utilized: posttraumatic stress disorder (posttraumatic stress OR post‐traumatic stress OR posttraumatic syndrome* OR post traumatic syndrome* OR PTSD OR PTSS OR trauma OR psychological distress OR psychotraumatology) and children (child* OR adolescent* OR teen* OR minor* OR youth* OR pediat* OR boy* OR girl*) and treatment (treatment* OR intervention* OR therapy OR psychotherapy OR exposure OR trial OR counseling). No restrictions were made regarding language.

All titles and abstracts of the search results were reviewed, and any that did not meet the inclusion criteria were removed. We then performed a full‐text review of the remaining publications. Two researchers independently reviewed all search results; any differences in opinion were discussed with the project's primary supervisor.

Quality assessments conducted by Hoppen and Morina (2020) were reviewed and updated where necessary for the present study. The quality of each study was assessed using the eight criteria proposed by Cuijpers et al. (2010; see the Supplementary Materials for the full quality assessment criteria). Namely, a study was considered to be of high quality if (a) participants meet the diagnostic criteria for PTSD, (b) a treatment manual was applied, (c) the providers who conducted the therapy were trained for the specific therapy, (d) treatment integrity was checked during the study, (e) data were analyzed with intent‐to‐treat analyses, (f) the study had a minimal level of statistical power to find significant effects of the treatment and included at least 50 patients in comparisons between the treatment and control groups, (g) the study reported that randomization was conducted by an independent party, and (h) outcome assessors were blinded to treatment condition (see Supplementary Materials for quality scores for each study). In line with Hoppen and Morina's (2020) protocol, we decided to label a trial as being of high quality if it reached a quality sum score of 7–8. Although two researchers independently assessed study quality, the researchers could not be blinded to Hoppen and Morina's (2020) ratings, potentially increasing the risk of bias in the quality assessment process. No ethical approval was gained as all data were obtained from previously published studies.

### Publication coding

Treatment outcome data and data on the trial therapists were extracted from each included publication along with any available data regarding any training providers received in the trial intervention and supervision providers received throughout the study. Therapist data were coded and categorized according to therapists’ professional background and educational attainment, the amount of training they received in the trial intervention, and the frequency of supervision provided (see Table 2 for therapist categorizations for each study). Therapists who received some training to provide the trial therapy but did not appear to have professional health care qualifications were defined as “lay therapists” (Lewin et al., 2010). Due to the variety of professional backgrounds reported for trial therapists, some were categorized together as “other therapists;” these included professionals such as CBT therapists, psychotherapists, and child‐advocacy service therapists. Two researchers independently extracted and coded data from each study, and any discrepancies were reviewed and discussed. Any disagreements regarding data categorization were discussed with the project's primary supervisor for resolution.

### Statistical analyses

Analyses were undertaken using *metaphor* in R (Vichtbauer, 2010). Means and standard deviations for postintervention PTSD symptoms were used to calculate Hedges’ *g* effect sizes for each study. When no standard deviation was reported, the standard error was computed using 95% confidence intervals; similarly, standard deviations were derived from confidence intervals if necessary.

For studies that utilized TF‐CBT in two experimental arms, results were pooled. When studies included both a passive and active control arm (Ertl et al., 2011; de Roos et al., 2017), the results from the passive control arm were used in the main analysis, but further sensitivity analyses were conducted using the active control arm to ensure this did not significantly impact the results (see Supplementary Materials). When further moderator analyses were conducted according to the type of control condition, both arms were included in each analysis, respectively. In one study (Cohen, 2011) standardized mean differences of change scores were reported instead of posttreatment data. Researchers have highlighted concerns about pooling these data in analyses (Deeks et al., 2020); therefore, the analysis was repeated as a sensitivity analysis with this paper removed to examine whether its inclusion affected the results (see Supplementary Materials). Follow‐up data were not included in our analyses because posttreatment data were arguably more pertinent to the research question, and there were inconsistencies in the timing and availability of follow‐up data reported across the included studies. Statistical heterogeneity was assessed using the *Q* test and quantified using the *I* ^2^ statistic. Random‐effects meta‐analyses were conducted along with subgroup analyses to compare the outcomes of interventions administered by different types of therapists.

## RESULTS

### Characteristics of the included studies

**FIGURE 1:**
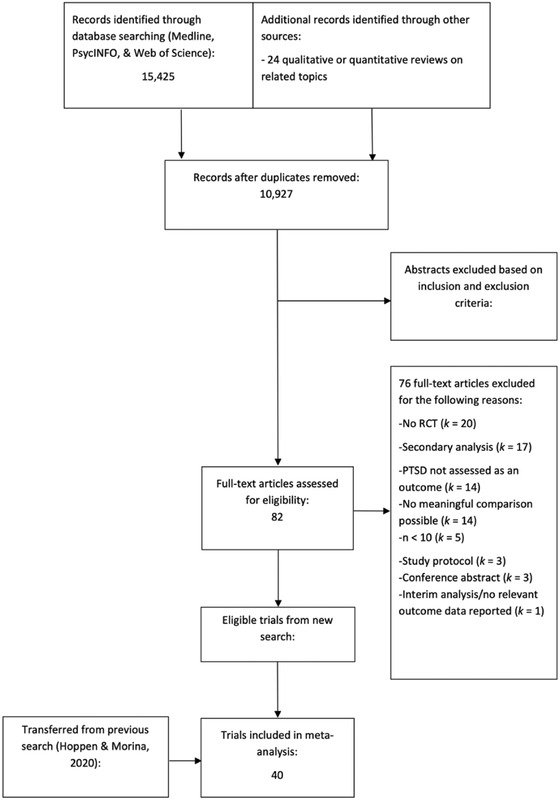
PRISMA flow diagram. Note. RCT = randomized controlled trial

Figure 1 illustrates the flow of publications through the systematic review. In total, 35 studies were transferred from Hoppen and Morina's (2020) review. Five further studies were identified as eligible following an updated search in May 2021; however. this resulted in eight additional comparisons, as one paper comprised four separate studies that were analyzed individually (Dorsey et al., 2020). An active control arm was used in 47.5% (*n* = 19) of the included studies.

### Quality of the included studies

As illustrated in the Supplementary Materials, overall, studies were of moderate quality, with a mean score of 5.8. No studies received a quality score between 0 and 2, three studies received a quality score of 3, and 17 studies were rated as high quality. The remainder of the studies had a quality rating of 4–6 (*n* = 20).

### Coding of therapists

Regarding therapist profession, 22.5% (*n* = 9) of the included studies employed either clinical psychologists or psychiatrists as trial therapists, 22.5% (*n =* 9) used counselors, 10.0% (*n =* 4) employed social workers, 30.0% (*n =* 12) used a variety of therapists from different professional backgrounds, and 12.5% (*n =* 5) studies used other therapist types (e.g., CBT therapists, psychotherapists). For one trial (Cohen et al., 2005), therapist profession was not reported.

Regarding therapist educational attainment, 22.5% (*n =* 9) of the included trials used therapists with a doctoral‐level education, 12.5% (*n =* 5) used therapists with a master's‐level education, 5.0% (*n =* 2) used therapists who had not engaged in any tertiary or higher education beyond secondary school level (i.e., school level), and 25.0% (*n =* 10) included therapists with a mixture of educational attainment; in 35.0% of the included studies (*n =* 14), therapist educational attainment was not reported. Lay therapists (i.e., therapists who did not appear to have formal health care qualifications) were used in 17.5% (*n =* 7) of studies, whereas the other 32 trials utilized therapists classified as being professional therapists (80.0%). In one trial, therapist status could not be ascertained (Cohen et al., 2005).

Among all included studies, 82.5% (*n =* 33) reported providing trial intervention–specific training to therapists. Of these trials, 37.5% (*n =* 15) reported providing training that was conducted over 3 days or longer, and the remainder reported training sessions of fewer than 3 days or did not report the amount of training provided (*n =* 18). Clinical supervision for trial therapists was reported in 82.5% (*n =* 33) of studies; of these, 45.0% (*n =* 18) provided supervision on a fortnightly basis or more frequently; the remainder either provided supervision less frequently than fortnightly (e.g., monthly; *n =* 2) or did not report supervision frequency (*n =* 16).

### Meta‐analysis

All studies were included in the meta‐analysis (*k* = 43); individual effect sizes for each study can be found in the Supplementary Materials. Consistent with Hoppen and Morina (2020), pooling effect sizes suggested that TF‐CBT interventions were more beneficial than combined passive and active control conditions at reducing PTSD symptoms, Hedges’ *g* = −0.70, 95% CI [−0.90, −0.51], *k* = 43. This was true even when restricting the included studies to those that included active control conditions. There was a large degree of heterogeneity between the studies, *I* ^2^ = 87.0%.

### Subgroup and moderator analyses

To address the primary research questions, subgroup and moderator analyses were conducted to investigate the influence of specific therapist factors on treatment outcomes. There were 40 studies included in the analysis comparing the effect of therapist professional background on treatment outcomes (see Table 1, 3); one study was excluded (Cohen et al., 2005), as no therapist characteristics were reported. The largest effect size, Hedges’ *g* = ‐1.11, was observed for clinical psychologists and psychiatrists; medium‐sized effects were observed for studies that used a mix of therapist types, social workers, and other types of therapists, although the effect size for social workers (*k* = 4) was not significant.

**TABLE 1:** Characteristics of the included publications

Publication	Type of intervention	Delivery format	Control treatment	Control group type	PTSD outcome measure	Country
Ahrens & Rexford (2002)	CPT group	Group	WL	PCC	PSS‐SR	United States
Auslander et al. (2017)	GAIN	group, PAR‐INV	TAU	ACC	CPSS	United States
Auslander et al. (2020)	CBITS	Group	UC	PCC	CPSS	United States
Barron et al. (2016)	TRT	Group	WL	PCC	CRIES	Palestine
Barron et al. (2020)	TRT	Group	WL	PCC	CRIES	Brazil
Catani et al. (2009)	KIDNET	Individual	MED	ACC	UPID	Sri Lanka
Celano et al. (2009)	Recovering from abuse program	Individual, PAR‐INV	TAU	ACC	CITES‐R	United States
Cohen et al. (2004)	TF‐CBT		CCT	ACC	K‐SADS	United States
Cohen et al. (2005)	TF‐CBT	individual, PAR‐INV	SC	ACC	TSCC	United States
Cohen et al. (2011)	TF‐CBT		CCT	ACC	K‐SADS	United States
Dawson et al. (2018)	TF‐CBT	individual, PAR‐INV	SC	ACC	UCLA PTSD‐RI	Indonesia
De Roos et al. (2011)	TF‐CBT	individual, PAR‐INV	EMDR	ACC	UCLA PTSD‐RI	The Netherlands
De Roos et al. (2017)	CBWT	Individual	EMDR & WL	ACC & PCC	CRTI	The Netherlands
Deblinger et al. (1996)	TF‐CBT	individual, child‐only, child/parent, parent only	TAU	ACC	K‐SADS	United States
Diehle et al. (2015)	TF‐CBT		EMDR	ACC	CAPS‐CA	Netherlands
Dorsey et al. (2020)	TF‐CBT	Group	UC	PCC	CPSS	Kenya, Tanzania
Ertl et al. (2011)	KIDNET	Individual	SC & WL	ACC & PCC	CAPS‐CA	Uganda
Foa et al. (2013)	PE	Individual	SC	ACC	CPSS‐I	United States
Gilboa‐Schechteman et al. (2010)	PE		PDP	ACC	CPSS	Israel
Goldbeck et al. (2016)	TF‐CBT	Individual, PAR‐INV	WL	PCC	CAPS‐CA	Germany
Hitchcock et al. (2022)	TF‐CBT		TAU	PCC	YC‐PTSD CL	United Kingdom
Jensen et al. (2014)	TF‐CBT individual, PAR‐INV	Individual, PAR‐INV	TAU	ACC	CAPS‐CA	Norway
Kameoka et al. (2020)	TF‐CBT		WL	PCC	K‐SADS	Japan
King et al. (2000)	TF‐CBT	Individual, child only, child/PAR‐INV	WL	PCC	ADIS	Australia
McMullen et al. (2013)	TF‐CBT	Group	WL	PCC	UCLA PTSD‐RI	Democratic Republic of the Congo
Meiser‐Stedman et al. (2017)	CT‐PTSD	Individual	WL	PCC	CPTSDI	United Kingdom
Murray et al. (2015)	TF‐CBT	Individual	TAU	ACC	UPID	Zambia
O'Callaghan et al. (2013)	TF‐CBT	Group, PAR‐INV	WL	PCC	UCLA‐PTSD‐RI	Democratic Republic of the Congo
O'Callaghan et al. (2015)	TF‐CBT	Group, PAR‐INV	MDT	ACC	UCLA‐PTSD‐RI	Democratic Republic of the Congo
Peltonen & Kangaslampi (2019)	NET	Individual	TAU	ACC	CRIES	Finland
Pityaratstian et al. (2015)	TF‐CBT	Group	WL	PCC	UCLA‐PTSD‐RI	Thailand
Rosner et al. (2019)	D‐CPT	Individual	WL	PCC	CAPS‐CA	Germany
Rossouw et al. (2018	PE	Group	SC	ACC	CPSS‐I	South Africa
Ruf et al. (2010)	KIDNET individual	Individual	WL	PCC	UPID	Germany
Schauer (2008)	KIDNET individual	Individual	MED	ACC	CAPS‐CA	Sri Lanka
Scheeringa et al. (2011)	CBT Individual, PAR‐INV	Individual, PAR‐INV	WL	PCC	PAPA	United States
Schottelkorb et al. (2012)	TF‐CBT	Individual	CCPT	ACC	UPID	United States
Shein‐Szydlo et al. (2016)	CBT‐TSC		WL	PCC	CPSS	Mexico
Smith et al. (2007)	TF‐CBT	Individual, PAR‐INV	WL	PCC	CAPS‐CA	United Kingdom
Stein et al. (2003)	TF‐CBT	Group	WL	PCC	CPSS	United States

*Note*: ADIS = Anxiety Disorders Interview Schedule; CAPS‐CA = Clinician‐Administered PTSD Scale for Children and Adolescents; CBT = cognitive behavior therapy; CBT‐TSC = CBT for trauma in street children; CCPT = child‐centred play therapy; CCT = child‐centred therapy; CITES‐R = Children's Impact of Traumatic Events Scales–Revised; CPSS = Child PTSD Symptom Scale; CPSS‐I = CPSS–Interview Version; CPT = cognitive processing therapy; CPTSDI = Child PTSD Inventor; CRIES = Children's Revised Impact of Event Scale; CRTI = Children's Responses to Trauma Inventory; CT = cognitive therapy; D‐CPT = developmentally adapted cognitive processing therapy; EMDR = eye movement desensitization and reprocessing; K‐SADS = Schedule for Affective Disorders and Schizophrenia for School‐Age Children; KIDNET = narrative exposure therapy for children; NET = narrative exposure therapy; PAPA = Preschool‐Age Psychiatric Assessment; PAR‐INV = parent involvement; PE = prolonged exposure; PDP = psychodynamic psychotherapy; SC = supportive counselling; TAU = treatment as usual; TF‐CBT = trauma‐focused cognitive behavior therapy; TSCC = Trauma Symptom Checklist for Children; UC = usual care; UCLA PTSD‐RI = University of California–Los Angeles PTSD Reaction Index; UPID = The University of California at Los Angeles PTSD Index; WL = waitlist.

**TABLE 2:** Categorization of trial therapists

Publication	Type of therapist	Therapist educational attainment^a^	CP/psychiatrist vs. other	Qualification: Master's degree or higher vs. other	Lay vs. professional
Ahrens & Rexford (2002)	Mixed	Doctoral	Mixed	Master's or higher	Professional
Auslander et al. (2017)	Other	NR	NR	NR	Professional
Auslander et al. (2020)	Mixed	Mixed	Mixed	Master's or higher	Professional
Barron et al. (2016)	Counselor	NR	Other	NR	Professional
Barron et al. (2020)	Mixed	NR	Mixed	Master's or higher	Professional
Catani et al. (2009)	Counselor	NR	Other	Other	Lay
Celano et al. (2009)	Mixed	Mixed	Mixed	Mixed	Professional
Cohen et al. (2004)	Mixed	Mixed	Mixed	Mixed	Professional
Cohen et al. (2005)	NR	NR	NR	NR	NR
Cohen et al. (2011)	Social worker	Master's	Other	Master's or higher	Professional
Dawson et al. (2018)	Counselor	School	Other	Other	Lay
De Roos et al. (2011)	Mixed	Mixed	Mixed	Master's or higher	Professional
De Roos et al. (2017)	CP/psychiatrist	Doctoral	CP/psychiatrist	Master's or higher	Professional
Deblinger et al. (1996)	Other	NR	Other	NR	Professional
Diehle et al. (2015)	Other	NR	Other	NR	Professional
Dorsey et al. (2020)	Counselor	NR	Other	Other	Lay
Ertl et al. (2011)	Counselor	NR	Other	Other	Lay
Foa et al. (2013)	Counselor	Master's	Other	Master's or higher	Professional
Gilboa‐Schechteman et al. (2010)	Mixed	Master's	Other	Master's or higher	Professional
Goldbeck et al. (2016)	Mixed	Mixed	Mixed	Master's or higher	Professional
Hitchcock et al (2022)	CP/psychiatrist	Doctoral	CP/psychiatrist	Master's or higher	Professional
Jensen et al. (2014)	Mixed	Mixed	Mixed	Mixed	Professional
Kameoka et al. (2020)	CP/psychiatrist	Doctoral	CP/psychiatrist	Master's or higher	Professional
King et al. (2000)	CP/psychiatrist	Doctoral	CP/psychiatrist	Master's or higher	Professional
McMullen et al. (2013)	Mixed	Mixed	Mixed	NR	Professional
Meiser‐Stedman et al. (2017)	CP/psychiatrist	Doctoral	CP/psychiatrist	Master's or higher	Professional
Murray et al. (2015)	Counselor	School	Other	Other	Professional
O'Callaghan et al. (2013)	Social worker	NR	Other	Other	Professional
O'Callaghan et al. (2015)	Mixed	NR	Other	Other	Lay
Peltonen & Kangaslampi (2019)	Mixed	Mixed	Mixed	Mixed	Professional
Pityaratstian et al. (2015)	CP/psychiatrist	Doctoral	CP/psychiatrist	Master's or higher	Professional
Rosner et al. (2019)	Other	Mixed	Other	Master's or higher	Professional
Rossouw et al. (2018	Other	NR	Other	Other	Lay
Ruf et al. (2010)	CP/psychiatrist	Doctoral	CP/psychiatrist	Master's or higher	Professional
Schauer (2008)	Counselor	NR	Other	NR	Professional
Scheeringa et al. (2011)	Social worker	Mixed	Other	Other	Professional
Schottelkorb et al. (2012)	Counselor	Master's	Other	Other	Lay
Shein‐Szydlo et al. (2016)	CP/psychiatrist	Master's	CP/psychiatrist	Master's or higher	Professional
Smith et al. (2007)	CP/psychiatrist	Doctoral	CP/psychiatrist	Master's or higher	Professional
Stein et al. (2003)	Social worker	NR	Other	NR	Professional

*Note*: CP = clinical psychologist; NR = not reported.

^a^ “School” refers to basic childhood education.

**TABLE 3:** Results on the efficacy of trauma‐focused cognitive behavioral therapy (TF‐CBT) interventions for child and adolescent posttraumatic stress disorder

Analysis	*k*	Hedge's *g*	95% CI	*I* ^2^ (%)	Moderator test coefficient (*Q* test statistic)	*p*
All studies	43	−0.70	[−0.90, −0.51]	86.7	−	−
By control condition					6.34	.012
ACC	19	−0.43	[−0.68, −0.18]	80.2		
PCC	24	−0.93	[−1.21, −0.65]	88.0		
By profession					−	−
CP/psychiatrist	9	−1.11	[−1.50, −0.72]	63.7		
Counselor	12	−0.41	[−0.67, −0.14]	81.0		
Social worker	4	−0.78	[−1.75, 0.19]	93.6		
Mixed	12	−0.83	[−1.32, −0.35]	92.4		
Other therapist	5	−0.68	[−1.00, −0.36]	41.6		
By educational attainment					−	−
Doctoral degree	9	−1.06	[−1.45, −0.68]	57.1		
Master's degree	6	−0.43	[−1.00, 0.14]	89.7		
School^a^	2	−0.55	[−1.62, 0.53]	93.3		
Mixed	9	−0.67	[−1.15, −0.19]	90.0		
CP/psychiatrist vs. other, ALL					4.07	.044
CP/psychiatrist	9	−1.11	[−1.50, −0.72]	63.7		
Other	24	−0.60	[−0.86, −0.35]	87.9		
CP/psychiatrist vs. other, ACC					NA	NA
CP/psychiatrist	1	0.27	[−0.16, 0.70]	NA		
Other	13	−0.44	[−0.81, −0.08]	85.3		
CP/psychiatrist vs. other, PCC					2.34	.126
CP/doctoral	9	−1.11	[−1.50, −0.72]	63.7		
Other	12	−.70	[−1.05, −0.36]	89.0		
Master's degree or higher vs. other, ALL					<0.01	.937
Master's degree or higher	17	−0.76	[−1.07, −0.45]	82.7		
Other	14	−0.75	[−1.16, −0.35]	91.2		
Master's degree or higher vs. other, ACC					1.16	.281
Master's degree or higher	5	−0.11	[−0.60, 0.39]	80.5		
Other	7	−0.56	[−1.17, 0.04]	89.5		
Master's degree or higher vs. other, PCC					0.26	.608
Master's degree or higher	13	−.93	[−1.24, −0.61]	75.6		
Other	8	−.82	[−1.36, −0.28]	92.1		
Lay vs. professional, ALL					1.91	.167
Lay	10	−0.47	[−0.88, −0.06]	88.4		
Professional	32	−0.80	[−1.03, −0.57]	85.8		
Lay vs. professional, ACC					0.16	.691
Lay	6	−0.47	[−1.16, 0.23]	87.3		
Professional	14	−0.35	[−0.60, −0.09]	77.0		
Lay vs. professional, PCC					5.40	.020
Lay	5	−0.38	[−0.77, 0.01]	84.1		
Professional	19	−1.10	[−1.41, −0.79]	84.4		

*Note*: ACC = active control condition; CP = clinical psychologist; PCC = passive control condition.

^a^ Refers to basic childhood education.

Similarly, in a further analysis of therapists’ educational background, the largest effect was observed for doctoral‐level therapists, Hedges’ *g* = −1.06. Small‐to‐medium effects were observed for therapists with a master's‐level education or a mixed educational background; however, for master's‐ and school‐level therapists, these effects were not significant.

A significant moderating effect was found when comparing the treatment outcomes of clinical psychologists and psychiatrists versus other professionals, *p* = .044; however, a further inspection revealed that studies that employed doctoral‐level therapists predominantly utilized a passive control arm; this significant effect was no longer apparent after controlling for this confound (i.e., including only studies that used a passive control group; see Table 1). Further moderator analyses demonstrated no significant differences between therapist profession or educational attainment (i.e., master's‐level qualification or above vs. other; lay vs. professional) and PTSD treatment outcomes regardless of whether all studies were included or studies were restricted to those with active or passive control conditions only (see Table 1). The one exception was when comparing lay therapists to professional therapists when a passive control condition was used. In this instance, professional therapists yielded larger effect sizes, Hedges’ *g* = −1.10, than lay therapists, Hedges’ *g* = −0.38. It is important to note that some comparisons involved very few studies; for example, only one included study that employed lay therapists utilized a passive control arm compared with six studies that utilized an active control. Note that data on supervision and therapy‐specific training provided to therapists could not be included in the present analyses due to limited data availability, which made it impossible to compare groups.

### Sensitivity analyses

Two sets of sensitivity analyses were undertaken. The first involved using the active rather than the passive arms of the Ertl et al. (2011) and de Roos et al. (2017) trials. The results are presented in the Supplementary Materials. The main change observed was that there was no longer a moderation effect of clinical psychologists or psychiatrists versus other professionals. Otherwise, the pattern of results was the same. The second sensitivity analysis involved removing the Cohen et al. 2011 study, which utilized an analysis of change scores (see Supplementary Materials). Again, the moderation effect of clinical psychologists and psychiatrists versus other professionals was no longer observed, but the moderation effect of lay versus professional therapies for passive control condition trials remained.

## DISCUSSION

The main goal of this paper was to collate and summarize the data regarding the professional characteristics of therapists who are delivering TF‐CBT interventions for children and youth in clinical trials; a secondary aim was to understand whether trial therapist factors influenced the efficacy of TF‐CBT in RCTs. The results of the current meta‐analysis suggested that TF‐CBT interventions performed better than control conditions at reducing PTSD symptoms, a finding consistent with numerous previous reviews in this area (e.g., Hoppen & Morina, 2020; John‐Baptiste Bastien et al., 2020; Mavranezouli et al., 2020). With respect to our research questions, there was no significant difference between therapist profession or therapist educational attainment on PTSD treatment outcome. In line with conclusions drawn from a previous study in this area (Pfeiffer et al., 2020), this result suggests that therapists from a variety of professional backgrounds are able to deliver TF‐CBT effectively. However, this finding must be set within the context of the current research being conducted within an artificial, RCT setting and, therefore, clinicians usually received a significant level of training and supervision—arguably more than is likely to be received in routine care. Although it is encouraging that a wide range of clinicians can deliver TF‐CBT effectively, these findings should be replicated within routine care and future meta‐analyses before generalizations can be made. Nevertheless, the current findings have potentially significant clinical and economic implications for health services. The suggestion that therapists with minimal professional training can elicit PTSD treatment outcomes comparable to doctoral‐level clinicians has potential implications for future service development and cost‐saving strategies. This seems particularly relevant for LMICS that lack professionals to provide psychological interventions (Morina et al., 2017). Of note, there is an increasing evidence base of collaborative work being conducted in LMICs to evaluate affordable interventions delivered by local providers within primary care settings in ways that are amenable to implementation within health structures. Thirteen trials included in the current review took place in LMICs (see Table 1). These encouraging developments will support the long‐term capacity needed to disseminate the interventions and promote upscaling to large numbers of children and adolescents in need (Singla et al., 2018).

The review demonstrated that across the 40 clinical trials examined, there was a large variation in the professional backgrounds of the therapists delivering TF‐CBT. Although there was variation in the amount and type of training and clinical supervision provided for trial therapists, we found that most therapists delivering TF‐CBT were provided with specific training in the treatment model and received regular clinical case supervision, which often occurred at least every 2 weeks. This finding does not corroborate with reports of supervision and training provision for therapists delivering TF‐CBT in routine care. Laksa and colleagues (2013) previously highlighted that there is frequently a disparity in the level of training and supervision provided in RCTs compared with the amount therapists receive in routine care, which could risk reducing the external validity of trial results. In terms of training, many therapists who deliver TF‐CBT in routine care have reported experiencing a lack of training in trauma approaches, which provides a barrier to delivering evidence‐based care for clients experiencing PTSD (Finch, Ford, Lombardo, & Meiser‐Stedman, 2020). Similarly, a survey completed in Canada by Czincz and Romano (2013) found that 78% of trauma therapists working with children had received no training in trauma approaches and 66% reported never receiving clinical supervision. These findings suggest that to deliver similar patient outcomes in routine care that treatments in clinical trials confer, more must be done to increase supervision frequency and improve training opportunities for therapists.

The RCTs included in the present review included therapists of vastly different professional backgrounds, and, as such, the current sample is likely to be representative of clinical practice, although the levels of training and supervision may differ. Moreover, as several of the included clinical trials were conducted in non‐Western countries, there is also potential for generalizability of these findings across cultural contexts and health care systems. Ideally, the present findings will be called upon to improve clinician confidence in delivering evidence‐based treatments for PTSD in children, particularly regarding aspects of treatment such as exposure, about which many clinicians report negative beliefs (e.g., Feeny et al., 2003; Gunter & Whittal, 2010; Zoellner et al., 2011) but are a component of many TF‐CBT treatments.

The current review utilized the search strategy implemented by Hoppen and Morina (2020) to investigate RCTs examining the efficacy of psychological treatments for children and youth with PTSD. Alternative methodologies could be considered and would likely reveal alternative perspectives. Utilizing a search strategy that incorporated “therapist factors” as a specific search term could broaden the variety of therapist factors considered beyond just the professional factors reported in RCTs. For example, future reviews regarding the effects of gender, age, or personality differences would be useful to inform the efficient matching of therapists and patients (Johns et al., 2019). It is possible that other therapist factors that could not be captured in the current review contributed to the outcomes; again, this highlights the importance of more thorough reporting of clinician factors within clinical trials. As more trials with detailed information on therapist characteristics accumulate, more powerful meta‐analyses and subanalyses will become feasible to address this important research question with more certainty.

To our knowledge, this was the first meta‐analysis on the efficacy of TF‐CBT interventions for PTSD in children and youth that assessed the potential influence of therapist characteristics on treatment efficacy. Our ability to differentiate between passive and active control conditions meant that this potential confound could be reduced, revealing that although a significant effect was found for doctoral‐level clinicians, this was no longer present once passive controls were removed. However, several limitations relate to the categorization of therapist factors and how these were operationalized. The variety of therapists’ professional backgrounds used in the examined RCTs made categorizing therapists challenging. Determining the educational level of clinicians involved was also hard to determine with certainty; for example, some social workers may have obtained a master's degree, whereas others may not have. This became additionally challenging when cultural differences were considered, as different routes to professional qualification are employed in different countries. In practice, we assumed therapist educational attainment reflected the most common route to qualification for a given profession in the United Kingdom, and any uncertainties were considered in consultation with the project's supervisor.

An additional challenge in conducting the review was the limited evidence base and lack of consistency in the therapist data reported by trial researchers; this limited the breadth of the review, as missing data meant that some comparisons included very few studies. Recent reviews on therapist effects have highlighted concerns that therapist characteristics are generally neglected in the clinical research process (Fjermestad et al., 2016; Karver et al., 2005). There is currently no clear guidance to researchers on the type of therapist data that should be reported in clinical trials, which provides challenges when looking to understand the effects these variables might have on treatment outcomes. Future research would benefit from comprehensive reporting of therapist characteristics, such as professional background, years of clinical experience, and educational history, as well as a thorough summary of the training provided during the trial and the nature of supervision provided. The consistent reporting of these factors would significantly improve the quality of future research into the influence therapists have on treatment outcomes and allow for useful clinical recommendations to be made as a result.

In conclusion, the results of this review support previous findings that TF‐CBT is an effective psychological treatment for PTSD in children and youth and suggests therapists with varied educational and professional backgrounds are able to produce positive results when delivering this treatment among this population within an RCT setting. However, limitations regarding the comprehensiveness of data availability necessitate the need for further research seeking to understand the effect that therapist factors may have on treatment outcomes for young people experiencing PTSD.

## OPEN PRACTICES STATEMENT

Data sharing does not apply to this article as no new data were created or analyzed in this study.

## AUTHOR NOTE

Richard Meiser‐Stedman trains mental health professionals in the use of trauma‐focused cognitive behavioral therapies for the treatment of posttraumatic stress disorder. The other authors have no conflicts of interest to declare.

## Supporting information

Appendix A: Reference list of included publications.
Appendix B: Quality ratings for all included trials (Morina & Hoppen, 2020, updated May 2021)
Appendix C: Quality items based on Cuijpers et al. (2010)
Appendix D: Forest plot showing effect sizes for included studies (random effects model)
Appendix E: Results on the efficacy of TF‐CBT interventions for child and adolescent PTSD – Ertl and De Roos active arms
Appendix F: Results on the efficacy of TF‐CBT interventions for child and adolescent PTSD ‐ Cohen (2011) removed
