# Supplementary material for: Associations between therapist factors and treatment efficacy in randomized controlled trials of trauma‐focused cognitive behavioral therapy for children and youth: A systematic review and meta‐analysis
Source: J Trauma Stress. 2022 Apr 27;35(5):1405–19. doi: 10.1002/jts.22840 (PMC9790492; doi:10.1002/jts.22840)
Supplement: Supplementary file 1 — Appendix A: Reference list of included publications. Appendix B: Quality ratings for all included trials (Morina & Hoppen, 2020, updated May 2021) Appendix C: Quality items based on Cuijpers et al. (2010) Appendix D: Forest plot showing effect sizes for included studies (random effects model) Appendix E: Results on the efficacy of TF‐CBT interventions for child and adolescent PTSD – Ertl and De Roos active arms Appendix F: Results on the efficacy of TF‐CBT interventions for child and adolescent PTSD ‐ Cohen (2011) removed [file JTS-35-1405-s001.docx]

**Appendix A: Reference list of included publications.**

Ahrens, J., & Rexford, L. (2002). Cognitive processing therapy for incarcerated adolescents with PTSD. *Journal of Aggression, Maltreatment & Trauma, 6*(1), 201–216. https://doi.org/10.1300/J146v06n01_10

Auslander, W., Mcginnis, H., Tlapek, S., Smith, P., Foster, A., Edmond, T., & Dunn, J. (2017). Adaptation and Implementation of a Trauma- focused Cognitive Behavioral Intervention for Girls in Child Welfare. *American Journal of Orthopsychiatry*, *87*(3), 206–215. https://doi.org/10.1037/ ort0000233

Barron, I., Abdallah, G., & Heltne, U. (2016). Randomized control trial of Teaching Recovery Techniques in rural occupied Palestine: effect on adolescent dissociation. *Journal of Aggression, Maltreatment & Trauma*, *25*(9), 955–973. https://doi.org/10.1080/10926771.2016.1231149

Catani, C., Kohiladevy, M., Ruf, M., Schauer, E., Elbert, T., & Neuner, F. (2009). Treating children traumatized by war and tsunami: A comparison between exposure therapy and meditation-relaxation in North-East Sri Lanka. *BMC Psychiatry, 9*, 22. <https://doi.org/10.1186/1471-244X-9-22>

Celano, M., Hazzard, A., Webb, C., & McCall, C. (1996). Treatment of traumagenic beliefs among sexually abused girls and their mothers: An evaluation study. *Journal of Abnormal Child Psychology, 24*(1), 1–17. https://doi.org/10.1007/BF01448370

Cohen, J. A., Deblinger, E., Mannarino, A. P., & Steer, R. A. (2004). A multisite, randomized controlled trial for children with sexual abuse-related PTSD symptoms. *Journal of the American Academy of Child and Adolescent Psychiatry, 43*(4), 393–402. https://doi.org/10.1097/01.chi.0000111364. 94169.f9

Cohen, J. A., Mannarino, A. P., & Knudsen, K. (2005). Treating sexually abused children: 1 year follow-up of a randomized controlled trial. *Child Abuse & Neglect, 29*(2), 135–145. https://doi.org/10.1016/j.chiabu.2004.12.005

Cohen, J. A., Mannarino, A. P., & Iyengar, S. (2011). Community treatment of posttraumatic stress disorder for children exposed to intimate partner violence a randomized controlled trial. *Archives of Pediatrics & Adolescent Medicine, 165*(1), 16–21. https://doi.org/10.1001/archpediatrics. 2010.247

Dawson, K., Joscelyne, A., Meijer, C., Steel, Z., Silove, D., & Bryant, R. A. (2018). A controlled trial of trauma-focused therapy versus problem- solving in Islamic children affected by civil conflict and disaster in Aceh, Indonesia. *Australian & New Zealand Journal of Psychiatry*, *52*(3), 253–261. https://doi.org/10.1177/0004867417714333

de Roos, C., Greenwald, R., den Hollander-Gijsman, M., Noorthoorn, E., van Buuren, S., & de Jongh, A. (2011). A randomized comparison of cognitive behavioral therapy (CBT) and eye movement desensitization and reprocessing (EMDR) in disaster-exposed children. *European Journal of Psychotraumatology, 2*, 5694. https://doi.org/10.3402/ejpt.v2i0.5694

de Roos, C., van der Oord, S., Zijlstra, B., Lucassen, S., Perrin, S., Emmelkamp, P., & de Jongh, A. (2017). Comparison of eye movement desensitization and reprocessing therapy, cognitive behavioral writing therapy, and wait-list in pediatric posttraumatic stress disorder following single-incident trauma: a multicenter randomized clinical trial. *Journal of Child Psychology and Psychiatry*, *58*(11), 1219–1228. https://doi.org/10. 1111/jcpp.12768

Deblinger, E., Lippmann, J., & Steer, R. (1996). Sexually abused children suffering posttraumatic stress symptoms: Initial treatment outcome findings. *Child Maltreatment, 1*(4), 310–321. https://doi.org/10.1177/1077559596001004003

Diehle, J., Opmeer, B. C., Boer, F., Mannarino, A. P., & Lindauer, R. J. L. (2015). Trauma-focused cognitive behavioral therapy or eye movement desensitization and reprocessing: What works in children with posttraumatic stress symptoms? A randomized controlled trial. *European Child & Adolescent Psychiatry, 24*(2), 227–236. https://doi.org/10.1007/s00787-014-0572-5

Ertl, V., Pfeiffer, A., Schauer, E., Elbert, T., & Neuner, F. (2011). Community-implemented trauma therapy for former child soldiers in Northern Uganda: a randomized controlled trial. *Journal of the American Medical Association*, *306*(5), 503–512. https://doi.org/10.1001/jama.2011.1060

Foa, E. B., McLean, C. P., Capaldi, S., & Rosenfield, D. (2013). Prolonged exposure vs supportive counselling for sexual abuse-related PTSD in adolescent girls a randomized clinical trial. *Journal of the American Medical Association, 310*(24), 2650–2657. https://doi.org/10.1001/jama.2013. 282829

Gilboa-Schechtman, E., Foa, E. B., Shafran, N., Aderka, I. M., Powers, M. B., Rachamim, L., & Apter, A. (2010). Prolonged exposure versus dynamic therapy for adolescent PTSD: A pilot randomized controlled trial. *Journal of the American Academy of Child and Adolescent Psychiatry, 49*(10), 1034–1042. https://doi.org/10.1016/j.jaac.2010.07.014

Goldbeck, L., Muche, R., Sachser, C., Tutus, D., & Rosner, R. (2016). Effectiveness of trauma-focused cognitive behavioral therapy for children and adolescents: A randomized controlled trial in eight German mental health clinics. *Psychotherapy and Psychosomatics*, *85*(3), 159–170. https://doi. org/10.1159/000442824

Jensen, T. K., Holt, T., Ormhaug, S. M., Egeland, K., Granly, L., Hoaas, L. C., & Wentzel-Larsen, T. (2014). A randomized effectiveness study comparing trauma-focused cognitive behavioral therapy with therapy as usual for youth. *Journal of Clinical Child and Adolescent Psychology, 43*(3), 356–369. https://doi.org/10.1080/15374416.2013.822307

King, N., Tonge, B., Mullen, P., Myerson, N., Heyne, D., Rollings, S., & Ollendick, T. (2000). Treating sexually abused children with posttraumatic stress symptoms: A randomized clinical trial. *Journal of the American Academy of Child and Adolescent Psychiatry, 39*(11), 1347–1355. https://doi.org/ 10.1097/00004583-200011000-00008

McMullen, J., O'Callaghan, P., Shannon, C., Black, A., & Eakin, J. (2013). Group trauma-focused cognitive-behavioral therapy with former child soldiers and other war-affected boys in the DR Congo: A randomized controlled trial. *Journal of Child Psychology and Psychiatry, 54*(11), 1231–1241. https://doi.org/10.1111/jcpp.12094

Meiser-Stedman, R., Smith, P., McKinnon, A., Dixon, C., Trickey, D., Ehlers, A., ... & Dalgleish, T. (2017). Cognitive therapy as an early treatment for post-traumatic stress disorder in children and adolescents: A randomized controlled trial addressing preliminary efficacy and mechanisms of action. *Journal of Child Psychology and Psychiatry*, *58*(5), 623–633. https://doi.org/10.1111/jcpp.12673

Murray, L. K., Skavenski, S., Kane, J. C., Mayeya, J., Dorsey, S., Cohen, J. A., ... & Bolton, P. A. (2015). Effectiveness of trauma-focused cognitive behavioral therapy among trauma-affected children in Lusaka, Zambia: a randomized clinical trial. *Journal of the American Medical Association Pediatrics*, *169*(8), 761–769. https://doi.org/10.1001/jamapediatrics.2015.0580

O'Callaghan, P., McMullen, J., Shannon, C., Rafferty, H., & Black, A. (2013). A randomized controlled trial of trauma-focused cognitive beha- vioral therapy for sexually exploited, war-affected Congolese girls. *Journal of the American Academy of Child and Adolescent Psychiatry, 52*(4), 359–369. https://doi.org/10.1016/j.jaac.2013.01.013

O'Callaghan, P., McMullen, J., Shannon, C., & Rafferty, H. (2015). Comparing a trauma focused and non trauma focused intervention with war affected Congolese youth: A preliminary randomized trial. *Intervention, 13*, 28–44. https://doi.org/10.1097/WTF.0000000000000054

Peltonen, K., & Kangaslampi, S. (2019). Treating children and adolescents with multiple traumas: a randomized clinical trial of narrative ex- posure therapy. *European Journal of Psychotraumatology*, *10*(1), e1558708. https://doi.org/10.1080/20008198.2018.1558708

Pityaratstian, N., Piyasil, V., Ketumarn, P., Sitdhiraksa, N., Ularntinon, S., & Pariwatcharakul, P. (2015). Randomized controlled trial of group cognitive behavioral therapy for post-traumatic stress disorder in children and adolescents exposed to tsunami in Thailand. *Behavioral and Cognitive Psychotherapy*, *43*(5), 549–561. https://doi.org/10.1017/S1352465813001197

Rosner, R., Rimane, E., Frick, U., Gutermann, J., Hagl, M., Renneberg, B., ... & Steil, R. (2019). Effect of developmentally adapted cognitive processing therapy for youth with symptoms of posttraumatic stress disorder after childhood sexual and physical abuse: A randomized clinical trial. *Journal of the American Medical Association Psychiatry*, *76*(5), 484–491. https://doi.org/10.1001/jamapsychiatry.2018.4349

Rossouw, J., Yadin, E., Alexander, D., & Seedat, S. (2018). Prolonged exposure therapy and supportive counselling for post-traumatic stress disorder in adolescents: Task-shifting randomized controlled trial. *The British Journal of Psychiatry*, *213*(4), 587–594. https://doi.org/10.1192/bjp. 2018.130

Ruf, M., Schauer, M., Neuner, F., Catani, C., Schauer, E., & Elbert, T. (2010). Narrative exposure therapy for 7- to 16-year-olds: A randomized controlled trial with traumatized refugee children. *Journal of Traumatic Stress, 23*(4), 437–445. https://doi.org/10.1002/jts.20548

Schauer, M. (2008). Trauma treatment for children in war build-up of an evidence-based large-scale mental health intervention in north-eastern Sri Lanka (Doctoral dissertation).

Scheeringa, M. S., Weems, C. F., Cohen, J. A., Amaya-Jackson, L., & Guthrie, D. (2011). Trauma-focused cognitive-behavioral therapy for posttraumatic stress disorder in three-through six year-old children: A randomized clinical trial. *Journal of Child Psychology and Psychiatry, 52*(8), 853–860. https://doi.org/10.1111/j.1469-7610.2010.02354.x

Schottelkorb, A. A., Doumas, D. M., & Garcia, R. (2012). Treatment for childhood refugee trauma: A randomized, controlled trial. *International Journal of Play Therapy, 21*(2),57. https://doi.org/10.1037/a0027430

Shein-Szydlo, J., Sukhodolsky, D. G., Kon, D. S., Tejeda, M. M., Ramirez, E., & Ruchkin, V. (2016). A Randomized Controlled Study of Cognitive–Behavioral Therapy for Posttraumatic Stress in Street Children in Mexico City. *Journal of Traumatic Stress*, *29*(5), 406–414. https://doi. org/10.1002/jts.22124

Smith, P., Yule, W., Perrin, S., Tranah, T., Dalgleish, T., & Clark, D. M. (2007). Cognitive-behavioral therapy for PTSD in children and adolescents: A preliminary randomized controlled trial. *Journal of the American Academy of Child and Adolescent Psychiatry, 46*(8), 1051–1061. https://doi.org/10. 1097/CHI.0b013e318067e288

Stein, B. D., Jaycox, L. H., Kataoka, S. H., Wong, M., Tu, W. L., Elliott, M. N., & Fink, A. (2003). A mental health intervention for schoolchildren exposed to violence — A randomized controlled trial. *Journal of the American Medical Association, 290*(5), 603–611. https://doi.org/10.1001/jama. 290.5.603

**Appendix B: Quality ratings for all included trials (Morina & Hoppen, 2020, updated May 2021)**

| Study | Q1. PTSD diagnosis | Q2. Treatment manual | Q3. Therapist training | Q4. Treatment integrity checks | Q5. ITT analysis | Q6. Statistical power | Q7. Random group allocation | Q8. Blind outcome assessments | Q sum score /8 |
| --- | --- | --- | --- | --- | --- | --- | --- | --- | --- |
| Ahrens & Rexford (2002) | 0 | 1 | 1 | 0 | 0 | 0 | 0 | 1 | 3 |
| Auslander et al. (2017) | 0 | 1 | 1 | 1 | 0 | 0 | 1 | 1 | 5 |
| Auslander et al. (2020) | 0 | 1 | 1 | 1 | 1 | 1 | 1 | 0 | 6 |
| Barron et al. (2016) | 0 | 1 | 1 | 1 | 1 | 1 | 1 | 1 | 7 |
| Barron et al. (2020) | 0 | 1 | 1 | 1 | 1 | 0 | 0 | 1 | 5 |
| Catani et al. (2009) | 0 | 1 | 1 | 1 | 1 | 0 | 1 | 1 | 6 |
| Celano et al. (1996) | 0 | 1 | 1 | 1 | 0 | 0 | 0 | 1 | 4 |
| Cohen et al. (2004) | 0 | 1 | 1 | 1 | 0 | 1 | 0 | 1 | 5 |
| Cohen et al. (2005) | 0 | 1 | 0 | 1 | 1 | 1 | 1 | 1 | 6 |
| Cohen et al. (2011) | 0 | 1 | 1 | 1 | 1 | 1 | 1 | 1 | 7 |
| Dawson et al. (2018) | 0 | 0 | 1 | 0 | 1 | 1 | 1 | 1 | 5 |
| De Roos et al. (2011) | 0 | 1 | 1 | 1 | 1 | 0 | 1 | 1 | 6 |
| De Roos et al. (2017) | 0 | 1 | 1 | 1 | 1 | 1 | 1 | 1 | 7 |
| Deblinger et al. (1996) | 0 | 1 | 1 | 1 | 0 | 0 | 0 | 0 | 3 |
| Diehle et al. (2015) | 0 | 1 | 1 | 1 | 1 | 0 | 1 | 1 | 6 |
| Dorsey et al. (2015) | 0 | 1 | 1 | 1 | 1 | 1 | 1 | 1 | 7 |
| Ertl et al. (2011) | 1 | 1 | 1 | 1 | 0 | 1 | 0 | 1 | 6 |
| Foa et al. (2013) | 0 | 1 | 1 | 1 | 1 | 1 | 1 | 1 | 7 |
| Gilboa- Schechteman et al. (2010) | 1 | 1 | 1 | 1 | 1 | 0 | 1 | 1 | 7 |
| Goldbeck et al. (2016) | 0 | 1 | 1 | 1 | 1 | 1 | 1 | 1 | 7 |
| Hitchcock et al. (2022) | 1 | 1 | 1 | 1 | 1 | 0 | 1 | 1 | 7 |
| Jensen et al. (2014) | 0 | 1 | 1 | 1 | 1 | 1 | 1 | 1 | 7 |
| Kameoka et al. (2014) | 0 | 1 | 1 | 1 | 1 | 0 | 1 | 1 | 6 |
| King et al. (2000) | 0 | 1 | 1 | 1 | 1 | 0 | 0 | 0 | 4 |
| McMullen et al. (2013) | 0 | 1 | 1 | 1 | 0 | 0 | 1 | 1 | 5 |
| Meiser-Stedman et al. (2017) | 1 | 1 | 1 | 1 | 0 | 0 | 0 | 1 | 5 |
| Murray et al. (2015) | 0 | 1 | 1 | 1 | 1 | 1 | 1 | 1 | 7 |
| O’Callaghan et al. (2013) | 0 | 1 | 1 | 1 | 1 | 1 | 1 | 1 | 7 |
| O’Callaghan et al. (2015) | 0 | 1 | 1 | 1 | 1 | 1 | 1 | 1 | 7 |
| Peltonen & Kangaslampi (2019) | 0 | 1 | 1 | 1 | 0 | 0 | 1 | 0 | 4 |
| Pityaratstian et al. (2015) | 1 | 1 | 1 | 0 | 0 | 0 | 0 | 1 | 4 |
| Rosner et al. (2019) | 1 | 1 | 1 | 1 | 1 | 1 | 1 | 1 | 8 |
| Rossouw et al. (2018 | 1 | 1 | 1 | 1 | 1 | 1 | 1 | 1 | 8 |
| Ruf et al. (2010) | 1 | 1 | 1 | 0 | 1 | 0 | 0 | 1 | 5 |
| Schauer (2008) | 1 | 1 | 1 | 1 | 1 | 0 | 1 | 1 | 7 |
| Scheeringa et al. (2011) | 0 | 1 | 0 | 1 | 0 | 0 | 1 | 0 | 3 |
| Schottelkorb et al. (2012) | 0 | 1 | 1 | 1 | 0 | 0 | 1 | 0 | 4 |
| Shein-Szydlo et al. (2016) | 1 | 1 | 1 | 1 | 0 | 1 | 1 | 1 | 7 |
| Smith et al. (2007) | 1 | 1 | 1 | 1 | 1 | 0 | 1 | 1 | 7 |
| Stein et al. (2003) | 0 | 1 | 1 | 1 | 0 | 1 | 1 | 1 | 6 |

**Appendix C: Quality items based on Cuijpers et al. (2010)**

| Q1 | All participants met diagnostic criteria for PTSD according to DSM or ICD criteria at baseline as assessed with a personal diagnostic interview, such as the SCID | 1. Positive 0. Negative / insufficient information |
| --- | --- | --- |
| Q2 | Use of treatment manual (i.e., published, or specifically designed for the study; in case of multiple experimental conditions all had to be were manual-based) | 1. Positive 0. Negative / insufficient information |
| Q3 | Therapists were specifically trained for the given therapy (i.e., particularly in advance to the study or only included trained therapists with substantial prior experience with the given therapy) | 1. Positive 0. Negative / insufficient information |
| Q4 | Treatment integrity was checked (i.e., by regular supervision and/or independent, systematic, quantitative analysis of protocol adherence measures) | 1. Positive 0. Negative / insufficient information |
| Q5 | Data analysed with intent-to-treat analysis (i.e., all relevant data were reported from ITT analyses) | 1. Positive 0. Negative / insufficient information |
| Q6 | Study had a minimal level of statistical power to find effects and included ≥50 participants in the comparison groups ((i.e., n of (smallest) experimental group + n of (smallest) comparison group ≥50)) | 1. Positive 0. Negative / insufficient information |
| Q7 | Independent and random sequence generation/allocation (i.e., independent person, computer-generated or sealed envelopes) | 1. Positive 0. Negative / insufficient information |
| Q8 | Blind outcome assessments (i.e., blinded assessors; if only outcome measure was self-report-based the item was rated as fulfilled) | 1. Positive 0. Negative / insufficient information |


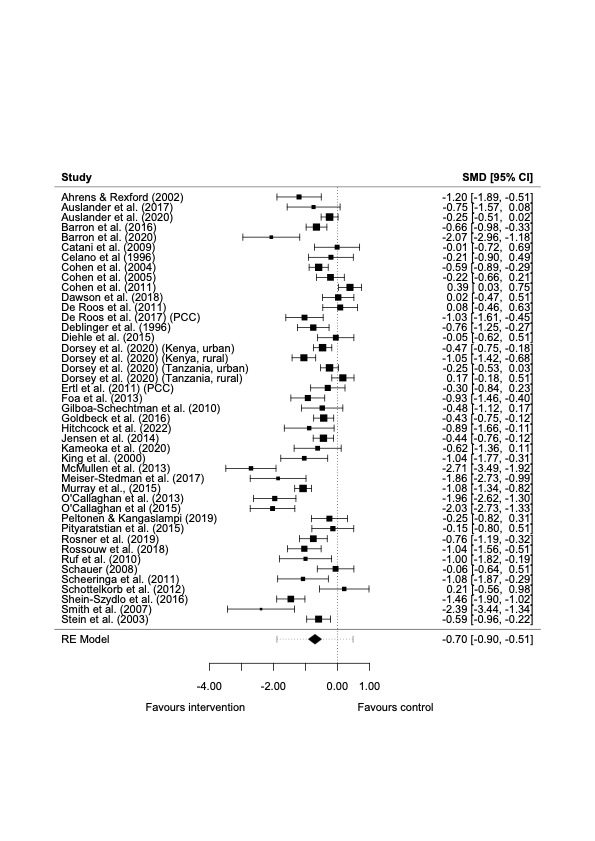
**Appendix D: Forest plot showing effect sizes for included studies (random effects model)**

**Appendix E: Results on the efficacy of TF-CBT interventions for child and adolescent PTSD – Ertl and De Roos active arms**

| Analysis | k | Hedge’s g | 95% CI | I^2^  (%) | Moderator test coefficient  (Q test statistic) | p |
| --- | --- | --- | --- | --- | --- | --- |
| *All studies* | 43 | -0.67 | -0.87, -0.46 | 88 | - | - |
|  |  |  |  |  |  |  |
| *By control condition:* |  |  |  |  | 8.61 | 0.003 |
| ACC | 21 | -0.37 | -0.61, -0.13 | 80 |  |  |
| PCC | 22 | -0.96 | -1.26, -0.66 | 89 |  |  |
|  |  |  |  |  |  |  |
| *By profession:* |  |  |  |  | - | - |
| CP /Psychiatrist | 9 | -0.96 | -1.49, -0.43 | 82 |  |  |
| Counsellor | 12 | -0.38 | -0.65, -0.10 | 82 |  |  |
| Social worker | 4 | -0.78 | -1.75, 0.19 | 94 |  |  |
| Mixed | 12 | -0.83 | -1.32, -0.35 | 92 |  |  |
| Other therapist | 5 | -0.68 | -1.00, -0.36 | 42 |  |  |
| *By educational level:* |  |  |  |  | - | - |
| Doctoral | 9 | -0.92 | -1.44, -0.40 | 79 |  |  |
| Master’s | 6 | -0.43 | -1.00, 0.14 | 90 |  |  |
| School | 2 | -0.55 | -1.62, 0.53 | 93 |  |  |
| Mixed | 9 | -0.67 | -1.15, -0.19 | 90 |  |  |
|  |  |  |  |  |  |  |
| *CP/Psychiatrist vs other, ALL* |  |  |  |  | 1.66 | 0.20 |
| CP/Psychiatrist | 9 | -0.96 | -1.49, -0.43 | 82 |  |  |
| Other | 24 | -0.59 | -0.85, -0.33 | 88 |  |  |
| *Master’s plus vs other, ALL* |  |  |  |  | 0.02 | 0.89 |
| Master’s plus | 17 | -0.68 | -1.01, -0.35 | 86 |  |  |
| Other | 14 | -0.73 | -1.14, -0.31 | 92 |  |  |
| *Lay vs Professional, ALL* |  |  |  |  | 1.68 | 0.19 |
| Lay | 10 | -0.44 | -.86, -0.01 | 89 |  |  |
| Professional | 32 | -0.76 | -1.00, -0.52 | 87 |  |  |
|  |  |  |  |  |  |  |

**Appendix F: Results on the efficacy of TF-CBT interventions for child and adolescent PTSD - Cohen (2011) removed**

| Analysis | k | Hedge’s g | 95% CI | I^2^  (%) | Moderator test coefficient  (Q test statistic) | p |
| --- | --- | --- | --- | --- | --- | --- |
| *All studies* | 42 | -0.73 | -0.93, -0.54 | 86 | - | - |
|  |  |  |  |  |  |  |
| *By control condition:* |  |  |  |  | 5.01 | 0.03 |
| ACC | 18 | -0.49 | -0.73, -0.25 | 76 |  |  |
| PCC | 24 | -0.93 | -1.21, -0.65 | 88 |  |  |
|  |  |  |  |  |  |  |
| *By profession:* |  |  |  |  | - | - |
| CP /Psychiatrist | 9 | -1.11 | -1.50, -0.72 | 64 |  |  |
| Counsellor | 12 | -0.41 | -0.67, -0.14 | 81 |  |  |
| Social worker | 3 | -1.18 | -2.00, 0.36 | 82 |  |  |
| Mixed | 12 | -0.83 | -1.31, -0.35 | 92 |  |  |
| Other therapist | 5 | -0.68 | -1.00, -0.36 | 42 |  |  |
| *By educational level:* |  |  |  |  | - | - |
| Doctoral | 9 | -1.06 | -1.45, -0.68 | 57 |  |  |
| Master’s | 5 | -0.61 | -1.16, -0.06 | 85 |  |  |
| School | 2 | -0.55 | -1.62, 0.53 | 93 |  |  |
| Mixed | 9 | -0.67 | -1.15, -0.19 | 90 |  |  |
|  |  |  |  |  |  |  |
| *CP/Psychiatrist vs other, ALL* |  |  |  |  | 3.59 | 0.06 |
| CP/Psychiatrist | 9 | -1.11 | -1.50, -0.72 | 64 |  |  |
| Other | 23 | -0.65 | -0.89, -0.40 | 86 |  |  |
| *CP/Psychiatrist vs other, ACC* |  |  |  |  | NA | NA |
| CP/Psychiatrist | 1 | 0.27 | -0.16, 0.70 | NA |  |  |
| Other | 12 | -0.52 | -0.89, -0.16 | 82 |  |  |
| *CP/Psychiatrist vs other, PCC* |  |  |  |  | 2.34 | 0.13 |
| CP/doctoral | 9 | -1.11 | -1.50, -0.72 | 64 |  |  |
| Other | 12 | -.70 | -1.05, -0.36 | 89 |  |  |
| *Master’s plus vs other, ALL* |  |  |  |  | 0.18 | 0.68 |
| Master’s plus | 16 | -0.83 | -1.11, -0.55 | 75 |  |  |
| Other | 14 | -0.75 | -1.16, -0.35 | 91 |  |  |
| *Master’s plus vs other, ACC* |  |  |  |  | .46 | 0.50 |
| Master’s plus | 4 | -0.25 | -0.80, 0.30 | 77 |  |  |
| Other | 7 | -0.56 | -1.17, 0.04 | 89 |  |  |
| *Master’s plus vs other, PCC* |  |  |  |  | 0.26 | 0.61 |
| Masters plus | 13 | -.93 | -1.24, -0.61 | 76 |  |  |
| Other | 8 | -.82 | -1.36, -0.28 | 92 |  |  |
| *Lay vs Professional, ALL* |  |  |  |  | 2.62 | 0.11 |
| Lay | 10 | -0.47 | -.88, -0.06 | 88 |  |  |
| Professional | 31 | -0.84 | -1.06, -0.61 | 83 |  |  |
| *Lay vs Professional, ACC* |  |  |  |  | 0.03 | 0.86 |
| Lay | 6 | -0.47 | -1.16, 0.23 | 87 |  |  |
| Professional | 13 | -0.42 | -0.66, -0.18 | 71 |  |  |
| *Lay vs Professional, PCC* |  |  |  |  | 5.40 | 0.02 |
| Lay | 5 | -0.38 | -0.77, 0.01 | 84 |  |  |
| Professional | 19 | -1.10 | -1.41, -0.79 | 84 |  |  |

*Note. ACC = active control condition; CP = clinical psychologist; Master’s plus = therapists educated to master’s level or above; PCC = passive control condition.*
